# *Primulina jiuyishanica* (Gesneriaceae), a new species from Hunan, China

**DOI:** 10.3897/phytokeys.162.53763

**Published:** 2020-10-07

**Authors:** Kun Liu, De-Chang Meng, Zhang-Jie Huang, Stephen Maciejewski, Zi-Bing Xin

**Affiliations:** 1 Jindong Forest Farm, Yongzhou 426191, China; 2 Guangxi Key Laboratory of Plant Conservation and Restoration Ecology in Karst Terrain, Guangxi Institute of Botany, Guangxi Zhuangzu Autonomous Region and Chinese Academy of Sciences, Guilin 541006, China; 3 Gesneriad Conservation Center of China (GCCC), Guilin Botanical Garden, Chinese Academy of Sciences, Guilin 541006, China; 4 The Gesneriad Society, 2030 Fitzwater Street, Philadelphia, PA 19146, USA

**Keywords:** Didymocarpoideae, flora of Hunan, *Primulina fimbrisepala*, taxonomy

## Abstract

*Primulina jiuyishanica* K. Liu, D.C. Meng & Z.B. Xin, a new species of Gesneriaceae from Hunan, China, is described and illustrated. The new species is morphologically similar to *Primulina fimbrisepala* (Hand.-Mazz.) Yin Z. Wang, but differs in its elliptic to broadly elliptic leaf blade with broadly cuneate base, peduncle densely pubescent with sparse glandular hairs, corolla throat with no purple spots inside, the yellow patch in the throat densely glandular-pubescent and pistil densely glandular-pubescent. Photographs and descriptions of the new species are provided below.

## Introduction

Since the original monotypic genus *Primulina* was redefined in 2011 (Wang et al. 2011; Weber et al. 2011), many taxa new to science have been published by taxonomists and researchers, making it the largest genus of the Chinese Gesneriaceae (Wen et al. 2019, 2020; Möller 2019). For instance, a total of 9 new species and one variety of *Primulina* were published in 2019, including *P. purpureokylin* F. Wen, Yi Huang & W.C. Chou, *P. persica* F. Wen, Yi Huang & W.C. Chou, *P. cerina* F. Wen, Yi Huang & W.C. Chou, *P. niveolanosa* F. Wen, S. Li & W.C. Chou, *P. leiyyi* F. Wen, Z.B. Xin & W.C. Chou (Li et al. 2019), *P. serrulata* R.B. Zhang & F. Wen (Jiang et al. 2019), *P. anisocymosa* F. Wen, Xin Hong & Z.J. Qiu (Hong et al. 2019), *P. chingipengii* W.B. Xu & K.F. Chung (Xu et al. 2019), *P. lianchengensis* B.J. Ye & S.P. Chen (Ye et al. 2019) and P. sichuanensis (W.T. Wang) Mich. Möller & A. Weber var. pinnatipartita H.H. Kong & L.H. Yang (Konget al. 2019). As this trend persists, more new species will likely be discovered in the near future (Möller 2019). *Primulina*, which mainly grows in limestone areas, are found only in southern and southwestern China and northern Vietnam (Möller et al. 2016). In all, 197 species and 27 varieties of *Primulina* exist at present, including 183 species and 27 varieties recorded from China and 21 species recorded from Vietnam (Hộ 2000; Burtt 2002; Weber et al. 2011; Möller et al. 2016; IPNI 2020; Wen et al. 2020).

In 2016, one of the authors (LK) discovered some plants in the Jiuyishan National Nature Reserve, Hunan Province, China, which possibly represented an undescribed species. Some living plants were mailed to the Gesneriad Conservation Center of China (GCCC) for observation and conservation. Those living individuals were introduced and cultivated in the gardens of the GCCC, and the lead author continuously monitored the population in the wild for several years. A detailed comparison of these specimens and living plant materials with the type specimens and protologues of known *Primulina* species revealed that these specimens neither fit the existing protologues nor conform to the type specimens of these species. Nevertheless, the inflorescence, shape and color of the corolla, stamens and staminodes are most similar to those of *P. fimbrisepala* (Hand.-Mazz.) Yin Z. Wang. It can be distinguished from the latter by a combination of several morphological characters of the leaf blade, peduncle, corolla throat and pistil. Thus, we confirmed that it represents a new species of *Primulina*, and described and illustrated it here. The description, illustration, information on ecology, phenology, and provisional conservation assessment by using IUCN categories and criteria (2019) of the proposed new species are also provided.

## Methods

The plant material for description was collected in the field at its type locality in 2017. Morphological observations and dissections of plant material of this new species were made under a stereoscopic microscope and measured and described using the terminology used by Wang et al. (1998). The literature examined included related monographs and papers (e.g., Wood 1974; Wang et al. 1998; Li and Wang 2004; Weber 2004; Haston and De Craene 2007; Chen et al. 2008; Wei et al. 2010). Specimens stored in herbaria in China, Vietnam, the United States and the United Kingdom (E, GH, HN, IBK, K, KUN, MO, PE, PH, US and VNMN) were examined.

## Taxonomic treatment

### Primulina jiuyishanica

Taxon classificationPlantaeLamialesGesneriaceae

K. Liu, D.C. Meng & Z.B. Xin sp. nov.

BD52323F-1773-5ECC-B2FF-C8CEF5AA0547

urn:lsid:ipni.org:names:77211929-1

Figure 1

#### Diagnosis.

*Primulina jiuyishanica* resembles *P. fimbrisepala* (Fig. 2) in having similar inflorescence and corolla color, but can be distinguished by its leaf blade being elliptic to broadly elliptic, base broadly cuneate (*vs.* blade ovate, broadly ovate to suborbicular, base cordate), peduncle densely pubescent with sparse glandular hairs (*vs.* eglandular-pubescent to appressed pilose), corolla throat with no purple spots inside (*vs.* corolla throat with few to many purple spots inside), the yellow patch in the throat densely glandular-pubescent (*vs.* densely eglandular-pubescent) and pistil densely glandular-pubescent (*vs.* eglandular-pubescent).

**Figure 1.**
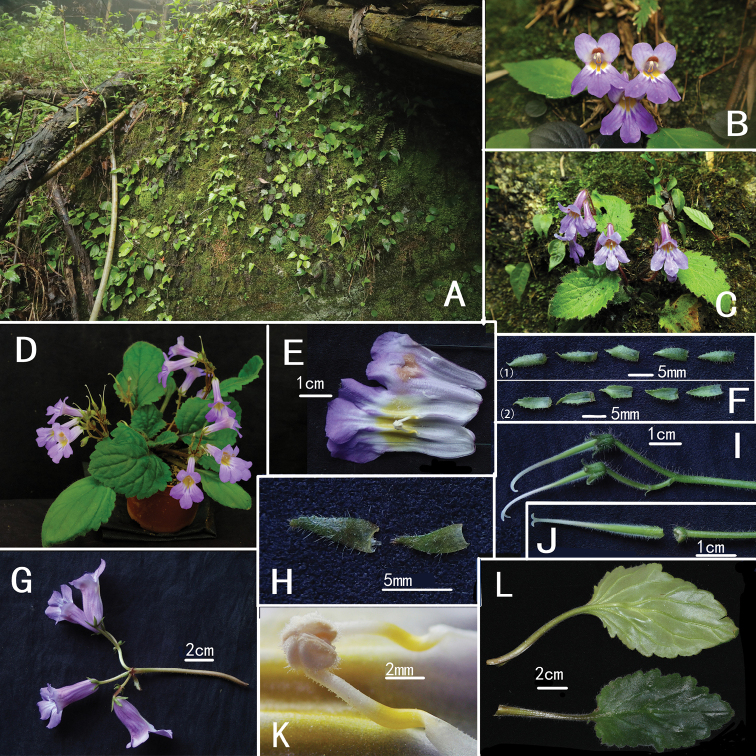
*Primulina jiuyishanica***A** habitat **B–C** habit with flowers **D** cultivated plant **E** opened corolla **F** (1) adaxial surfaces of calyx lobes (2) abaxial surfaces of calyx lobes **G** inflorescence **H** bracts **I** inflorescence after the corolla shedding **J** dissected ovary, disc and pedicel **K** stamens **L** abaxial and adaxial surfaces of leaf blades (Photographed by Fang Wen).

#### Type.

China. Hunan Province, Yongzhou City, Ningyuan County, Jiuyishan National Nature Reserve, growing on a granite rock in the forest in a damp and moist valley, alt. 900–1300 m, 14 April 2017, *Kun Liu 20170414-01* (holotype: IBK!; isotypes: IBK!, KUN!).

#### Description.

***Herbs*** perennial, rhizomatous. ***Stem*** subterete, ca. 6 cm long, ca. 2 cm in diameter. ***Leaves*** 4–6, basal, opposite; leaf blade thickly chartaceous, elliptic to broadly elliptic, 6–9 × 6–7 cm, margin conspicuously serrate, lateral veins 4–5 on each side, abaxially conspicuous, apex obtuse, base broadly cuneate, oblique or slightly asymmetric, adaxially densely pubescent with sparse glandular hairs, abaxially densely pubescent. ***Petiole*** flattened, 4–5 cm long, ca. 0.5 cm wide, densely villous. ***Cymes*** 1–5 or more, axillary, 1–2-branched, 2–14-flowers per cyme; ***peduncles*** 6–8 cm long, 2.5–3 mm in diameter, densely pubescent with sparse glandular hairs; ***pedicel*** 1–2.3 cm long, ca. 2 mm in diameter, densely pubescent with sparse glandular hairs; ***bracts*** 2, opposite, narrowly lanceolate, 1.1–1.2 cm long, 2–3.5 mm wide, sparse hydathodes, both surfaces sparsely pubescent; ***bracteole*** 1, lanceolate, 3–5 mm long, 1–2 mm wide, sparse hydathodes, both surfaces sparsely pubescent. ***Calyx*** 5-parted to near base, lobes narrowly lanceolate, 5–7 × 2–3 mm, sparse hydathodes on each side; apex acute, outside densely pubescent, inside subglabrous. ***Corolla*** pink to bluish violet, 4–4.5 cm long, 2.6–3 cm wide; ***corolla tube*** funnelform, 2.5–3 cm long, 1.2–1.5 cm in diameter, outside glandular-pubescent, inside glabrous; with two distinct longitudinal ridges on the corolla tube floor; a yellow patch at corolla throat extends to the middle of the corolla tube, densely glandular-pubescent; limb distinctly 2-lipped, adaxial lip 2-lobed, lobes broadly ovate, abaxial lip 3-lobed, middle lobe narrowly orbiculate or broadly ovate, lateral lobes oval or oblong. ***Stamens*** 2, adnate to ca. 1.4 cm above the base of the corolla tube; 9–10 mm long, terete, geniculate near middle, knee greenish yellow, the rest white, sparsely glandular-pubescent; anthers fused by the entire adaxial surfaces, abaxially densely whitish pubescent; ***staminodes*** 3, lateral ones ca. 4 mm long, adnate to ca. 9 mm above the base of the corolla tube, terete, apically capitate, the middle one ca. 0.9 mm long, adnate to ca. 1.5 mm above the base of the corolla tube. ***Disc*** annular, ca. 1 mm high, margin repand. Pistil 3.1–3.6 cm long; ovary cylindrical, 1.2–1.6 cm long, ca. 4.5 mm in diameter, densely glandular-pubescent to glandular-puberulent; ***style*** 1.5–1.6 cm long, 1–1.5 mm in diameter, densely glandular-pubescent to glandular-puberulent; ***stigma*** chiritoid, lower lobe ca. 1 mm wide, divided, lobes ca. 4 mm long. ***Capsule*** linear, 5–5.5 cm long, densely glandular-pubescent to glandular-puberulent.

**Figure 2.**
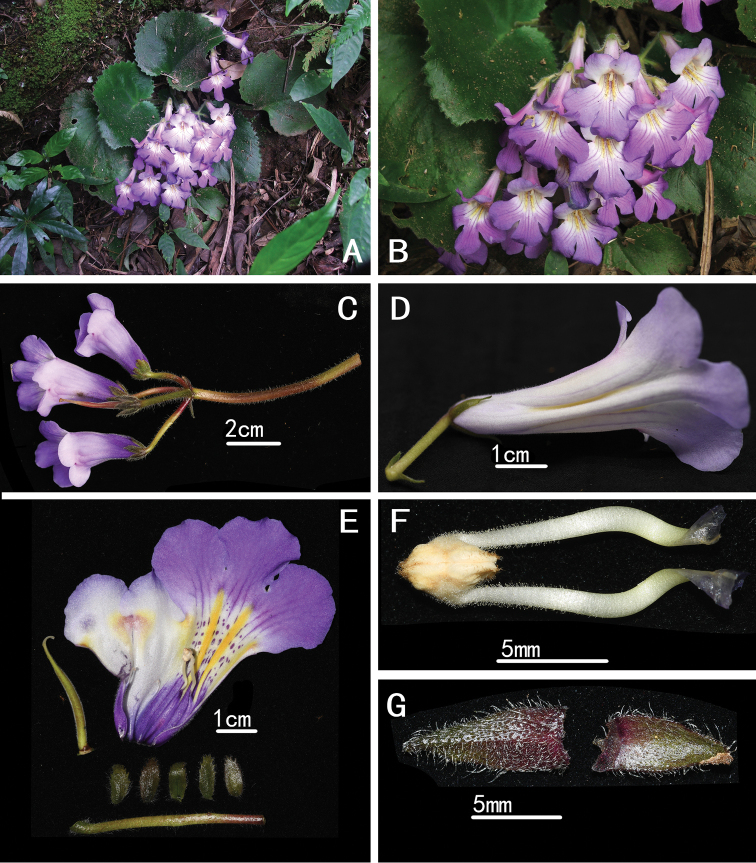
*Primulina fimbrisepala* (**A–G**) **A** habitat **B** habit in wild with flowers **C** inflorescence **D** two distinct long longitudinal ridges on the corolla tube floor **E** opened corolla **F** stamens **G** bracts. (**A–B**: Photographed by Xiao-Ning You; **C–G**: Photographed by De-Chang Meng).

#### Distribution and habitat.

We found eight populations in different places of Jiuyishan National Nature Reserve for the new species through several field investigations. It grows on granite rocks, in association with *Pileostegia viburnoides* Hook. f. & Thoms., *Hedera sinensis* (Tobl.) Hand.-Mazz, *Euonymus actinocarpus* Loes., *Viola kosanensis* Hayata, *Lysimachia congestiflora* Hemsl., *Goodyera biflora* (Lindl.) Hook. f., *Phyllagathis cavaleriei* Guillaum. and *Dryopteris* Adans. spp. in the forest in a damp and moist valley.

#### Phenology.

Flowering from April to May; fruiting from June to August.

#### Etymology.

The specific epithet is derived from the type locality, Jiuyishan National Nature Reserve, Hunan Province, China.

#### Vernacular name.

九嶷山报春苣苔(Chinese name); jiǔ yí shān bào chūn jù tái (Chinese pronunciation).

#### Conservation status.

The EOO and AOO of *Primulina jiuyishanica* are 54.28 km^2^ and 5.2 km^2^ respectively. So far, only eight populations of this species were found located in a nature reserve. However, we believe that more populations will be found in the future, and the EOO and AOO might increase. The eight populations have in total more than 3000 mature plants in the type locality, and additionally many seedlings were found. The plants are well protected in the nature reserve. According to the guidelines for using the IUCN Red List Categories and Criteria (IUCN 2019), the new species should be assessed as of Least Concern (LC).

#### Note.

In figure 2, A and B were published in Wei et al. 2010, page 374, 375, under the name of *Chirita juliae* Hance, now *Primulina juliae* (Hance) Mich. Möller & A. Weber. In fact, A and B are *P. fimbrisepala* (Hand.-Mazz.) Yin Z. Wang.

## Supplementary Material

XML Treatment for Primulina jiuyishanica
